# Methylphenidate-associated myopathy

**DOI:** 10.1186/1546-0096-10-S1-A96

**Published:** 2012-07-13

**Authors:** Ana Beatriz Vargas, Blanca ERG Bica

**Affiliations:** 1 Hospital Universitário Clementino Fraga Filho - Universidade Federal do Rio de Janeiro, Rio de Janeiro, Brazil

## Purpose

Methylphenidate (MP) (Ritalin®, Concerta®) is a drug commonly used for the treatment of children affected by attention-deficit hyperactivity disorder (ADHD). Literature shows some cases of cardiovascular side-effects, including arrhythmia and sudden death, but we failed to find a report of associated skeletal-muscle manifestation.

## Methods

We describe a case of a 13 yo girl diagnosed with ADHD since early childhood, in MP use for the past 3 years. She complained about a mild discomfort of proximal muscles of the lower limbs and cramps for the previous 3 months. Routine exams revealed increased aminotransferases levels (AST 192 UI/L; ALT 222 UI/L), suggesting hepatotoxicity. New laboratory tests were ordered, showing a CPK level of 18300 UI/L, AST 393 and ALT 330. The MP use became intermittent while the investigation was performed. Two weeks later, AST was 80 and ALT 166. A thorough investigation showed negative viral serology (HAV, HBV, HCV, HIV, CMV and EBV); negative autoantibodies (FAN, anti-LKM, anti-smooth muscle, anti-TPO and anti-tireoglobulin); normal hemogram; negative inflammatory markers (ESR and PCR) and a normal lower limbs MRI. An ECG revealed an altered antero-septal repolarization and echocardiogram was normal. Twenty days after, a new lab evaluation revealed a decreasing level of CPK (9670). One month after the complete withdrawal of the MP, the myalgia and cramps were no more present and CPK levels decreased to 3160mg/dl.

## Results

The decreasing levels of the muscle enzymes and clinical normalization after the drug discontinuation strongly suggested a cause-effect association.

## Conclusion

As the use of MP has increased so much in the past decade, new side effects have been related to this drug. This case may be the first in the literature to show the association of proximal myopathy to it, and the use of MP may become an uncommon differential diagnosis for some cases of proximal myopathy in children and teenagers.

## Disclosure

Ana Beatriz Vargas: None; Blanca E. R. G. Bica: None.

